# Development and comparison of RNA-sequencing pipelines for more accurate SNP identification: practical example of functional SNP detection associated with feed efficiency in Nellore beef cattle

**DOI:** 10.1186/s12864-020-07107-7

**Published:** 2020-10-08

**Authors:** S. Lam, J. Zeidan, F. Miglior, A. Suárez-Vega, I. Gómez-Redondo, P. A. S. Fonseca, L. L. Guan, S. Waters, A. Cánovas

**Affiliations:** 1 Centre for Genetic Improvement of Livestock, Department of Animal Biosciences, University of Guelph, 50 Stone Road E, Guelph, Ontario N1G2W1 Canada; 2 Spanish National Institute for Agriculture and Food Research and Technology, Carretera de La Coruña, 28040 Madrid, Spain; 3 Department of Agriculture, Food & Nutritional Science, University of Alberta, Edmonton, Alberta T6H 2P5 Canada; 4 Teagasc, Animal & Grassland Research and Innovation Centre, Grange, Dunsany, Co. Meath, C15 PW93 Ireland

**Keywords:** Feed efficiency, Bovine, RNA-Seq, Single nucleotide polymorphisms (SNPs), Transcriptomics

## Abstract

**Background:**

Optimization of an RNA-Sequencing (RNA-Seq) pipeline is critical to maximize power and accuracy to identify genetic variants, including SNPs, which may serve as genetic markers to select for feed efficiency, leading to economic benefits for beef production. This study used RNA-Seq data (GEO Accession ID: PRJEB7696 and PRJEB15314) from muscle and liver tissue, respectively, from 12 Nellore beef steers selected from 585 steers with residual feed intake measures (RFI; *n* = 6 low-RFI, *n* = 6 high-RFI). Three RNA-Seq pipelines were compared including multi-sample calling from i) non-merged samples; ii) merged samples by RFI group, iii) merged samples by RFI and tissue group. The RNA-Seq reads were aligned against the UMD3.1 bovine reference genome (release 94) assembly using STAR aligner. Variants were called using BCFtools and variant effect prediction (VeP) and functional annotation (ToppGene) analyses were performed.

**Results:**

On average, total reads detected for Approach i) non-merged samples for liver and muscle, were 18,362,086.3 and 35,645,898.7, respectively. For Approach ii), merging samples by RFI group, total reads detected for each merged group was 162,030,705, and for Approach iii), merging samples by RFI group and tissues, was 324,061,410, revealing the highest read depth for Approach iii). Additionally, Approach iii) merging samples by RFI group and tissues, revealed the highest read depth per variant coverage (572.59 ± 3993.11) and encompassed the majority of localized positional genes detected by each approach. This suggests Approach iii) had optimized detection power, read depth, and accuracy of SNP calling, therefore increasing confidence of variant detection and reducing false positive detection. Approach iii) was then used to detect unique SNPs fixed within low- (12,145) and high-RFI (14,663) groups. Functional annotation of SNPs revealed positional candidate genes, for each RFI group (2886 for low-RFI, 3075 for high-RFI), which were significantly (*P* < 0.05) associated with immune and metabolic pathways.

**Conclusion:**

The most optimized RNA-Seq pipeline allowed for more accurate identification of SNPs, associated positional candidate genes, and significantly associated metabolic pathways in muscle and liver tissues, providing insight on the underlying genetic architecture of feed efficiency in beef cattle.

## Background

High-throughput RNA-Sequencing (RNA-Seq) technology is widely used to detect and quantify expressed transcripts, novel transcript discovery and analyze differential gene expression and alternative splicing in a biological sample [1–3]. In addition to these applications, RNA-Seq can detect functional genetic variants such as single nucleotide polymorphisms (SNPs), which are restricted to the expressed portion of the genome and represent a large amount of genetic variation in the genome [4, 5]. SNP based genetic markers are useful due to their high abundance in the cattle genome [6, 7].

RNA-Seq experiments in livestock studies have identified significant SNPs in candidate genes associated with metabolic pathways that may play a role in the regulation of production traits [4, 8–12]. This has resulted in an improved understanding of the genetic architecture and a reduction in genome complexity of important traits such as feed efficiency, health, fertility, and meat quality traits in beef cattle [4, 8, 13–15]. More specifically, the study of genetic variants that may serve as markers to select for feed efficiency or residual feed intake (RFI) may help lead to the genetic improvement of feed efficiency and result in economic and environmental benefits for beef production, as feed costs represent approximately 70% of livestock production expenses [16].

Although SNP identification for genetic markers has served as a powerful tool in genomics, the ability to better understand the relationship between genotype and phenotype relies on the accuracy of analysis to detect genomic variation. Studies have previously compared methods for genotype calling software such as GATK, Samtools, SNPiR, CLC Bio Genomics Workbench using RNA-Seq data [4, 17–22], as well as variant calling using whole genome sequence data [23, 24]. Additionally, Brouard et al. [25] demonstrated the improved sensitivity of joint genotype calling using GATK compared to individual calling; however, studies have not compared merging approaches of RNA-Seq data across multiple samples per group and tissues.

Therefore, the evaluation of RNA-Seq pipelines to identify variants across different phenotypic or genotypic groups that include samples from multiple tissues has not been evaluated and strategies for the use and merging of RNA-Seq data from multiple samples and tissues for optimized power and accuracy remain limited. Optimized RNA-Seq analysis approaches can be applied for SNP discovery to detect SNPs that may serve as functional genetic markers and be used in selection strategies to improve economically relevant traits in livestock.

The aim of this study was to compare three RNA-Seq sample merging pipelines for SNP identification to determine the most optimized and accurate pipeline based on study experimental design. The approach considered as the most optimized and accurate approach for SNP detection using RNA-Seq data was then used to identify functional SNPs associated with feed efficiency in Nellore beef cattle to improve the understanding of the biology and metabolic pathways underlying genetic markers that may influence the function and regulation of feed efficiency in beef cattle. The objectives of this study were to 1) compare three RNA-Seq pipelines using samples from two divergent groups for feed efficiency (i.e., low- and high-RFI) and two tissues (i.e., liver and muscle) including multi-sample calling from: i) non-merged samples, ii) merged samples for low-RFI and merged samples for high-RFI for each tissue (merged by RFI group), iii) merged samples for low- and high-RFI for both tissues (merged by RFI and tissue group), 2) determine the pipeline with maximized accuracy and power for SNP detection and apply it to identify unique SNPs, and associated functional information, fixed within high or low feed efficient Nellore beef steers.

## Results and discussion

In this study, three RNA-Seq pipeline approaches and their variant calling results were compared. The most optimized approach was then applied to perform a more accurate SNP detection for genetic markers associated with feed efficiency in beef cattle. The number of total reads, total uniquely mapped reads, and percentage of uniquely mapped reads is reported in (Additional file 1). Overall, the number of uniquely mapped reads (number of reads that individually mapped to one location) identified in muscle tissue (205,269,868) were observed to be greater than that detected in liver tissue (87,466,593) (Additional file 1). This may have resulted in a lower number of total SNPs detected in liver compared to muscle in both the non-merged and merged samples approaches (Table 1).

**Table 1:** Summary of total SNPs detected using bcftools for each approach scenario used for comparisons

Approach scenario description	n	Total SNPs before filtering	Total SNPs after filtering	Percentage of SNPs passing all filters (%)
Approach i) non-merged samples				
Liver tissue	6	626,460	258,120	41.20
Muscle tissue	6	940,143	396,705	42.20
Liver and Muscle tissue	12	1,205,664	511,092	42.39
Approach ii) merged samples by RFI group				
Liver tissue	6	521,588	197,309	37.82
Muscle tissue	6	770,685	296,169	38.43
Liver and Muscle tissue	12	1,005,696	388,322	38.61
Approach iii) merged samples by RFI and tissue group				
Liver and Muscle tissue	12	1,048,370	416,216	39.70

i) non-merged samples; ii) merged samples by group for low-RFI and merged samples for high-RFI for each tissue, iii) merged samples by group and tissue for low- and high-RFI for both tissues

n = total number of samples

Table 2 displays all merging and non-merging approaches and the total SNPs identified before and after applying quality filters. On average, the percentage of SNPs that passed all quality filters for all approaches was 40.05 ± 1.88%. The high percentage of overlapping SNPs between low- and high-RFI groups was on average 77.54% (Table 2). Therefore, the majority of SNPs are shared between both extreme RFI groups, allowing for a reduced number of SNPs (less than 30%) that may be more important in the regulation of feed efficiency. A higher number of total SNPs were identified in the non-merging method compared to all other merging approaches. This may be due to an increase in detection of rare variants (i.e., variants detected in a small subset of the animals) (Table 1).

**Table 2:** Results of approach comparisons showing total SNP identified unique within approach and shared between both approaches

	Approach comparisons			
	Approach i) v.s. Approach ii)			
	Liver Non-merged v.s. Liver Merged by RFI group			
	Non-merged	Shared	Merged by RFI	Total
Total number of SNPs	61,158	196,962	347	258,467
Percentage of SNPs	23.66	76.20	0.13	
	Approach i) v.s. Approach ii)			
	Muscle Non-merged v.s. Muscle Merged by RFI group			
	Non-merged	Shared	Merged by RFI	Total
Total number of SNPs	101,047	295,658	511	397,216
Percentage of SNPs	25.44	74.43	0.13	
	Approach i) v.s. Approach iii)			
	Liver and Muscle Non-merged v.s. Liver and Muscle Merged by RFI group and Tissues			
	Non-merged	Shared	Merged by tissues	Total
Total number of SNPs	120,301	390,791	25,425	536,517
Percentage of SNPs	22.42	72.84	4.74	
	Approach ii) v.s. Approach iii)			
	Liver and Muscle Merged by RFI group v.s. Liver and Muscle Merged by RFI group and Tissues			
	Merged by RFI	Shared	Merged by tissues	Total
Total number of SNPs	14,699	373,623	42,593	430,915
Percentage of SNPs	3.41	86.70	9.88	

i) non-merged samples; ii) merged samples by group for low-RFI and merged samples for high-RFI for each tissue, iii) merged samples by group and tissue for low- and high-RFI for both tissues

### Comparison of RNA-Seq merging approaches for more accurate SNP detection

Currently, much of the variant calling studies have been performed using whole genome sequence data [23, 24]. Use of whole genome data allows for the identification of variants in an individual or group of individuals, allowing for detection of potential causal variants in the whole genome that may be associated with a trait of interest. In addition, when using genome sequence data, more non-coding variants can be identified as they are more present in the genome compared to coding variants [26]. In contrast, evaluation of the transcriptome using RNA-Seq allows for detection of variants within coding regions which may provide functional information regarding a trait of interest [4]. Additionally, RNA-Seq allows for the measure of differentially expressed genes between extreme phenotypic groups or treatments, however, relevancy of RNA-Seq data and expression profiles is dependent on the tissue, time-point, and condition analyzed [27]. With proper experimental design, the study of expression profiles and detection of genetic variants using RNA-Seq can provide a better understanding of the impact of genetic variants in tissues at specific time-points and conditions. Additionally, with a sufficient sample size, identification of expression QTL (eQTL) is possible, in order to evaluate the impact of genetic variants on the expression levels of genes associated with complex traits [9, 28–31]. With appropriate experimental design and optimized RNA-Seq pipelines, RNA-Seq can provide important information underlying the functional genetic mechanisms underlying a trait, such as genetic variants, key regulatory genes, and biological pathways. This study performed several analyses to compare RNA-Seq pipeline approaches in aim to optimize variant calling using RNA-Seq technology for the investigation of the underlying genetics of livestock traits.

To compare the overlap of the SNPs detected by the various approaches, we determined the total number and percentage of SNPs identified as shared or unique across the approaches being compared (Table 2). When observing the first comparison in Table 2, results revealed that when comparing Approach i) (non-merged) and Approach ii) (merged by RFI group) for liver, the majority of SNPs were shared (76.20%) between both approaches. A considerable number of SNPs detected by the Approach i) (23.66%) that were unique to this approach and were not detected by Approach ii), while very few SNPs (0.13%) were found to be uniquely detected by Approach ii). Similar results were found in the second comparison of Table 2 which performed the same comparison in muscle tissue. In the third comparison of Table 2, where Approach i) is compared with Approach iii) (merged by RFI and tissue groups), similar results were found. In this comparison, the majority of SNPs found shared (72.84%), 22.42% SNPs found unique to Approach i), and 4.74% SNPs found unique to Approach ii). The last comparison in Table 2 compared Approach ii) and Approach iii), where a greater overlap of SNPs was detected (86.70%), with 3.41% of SNPs found unique to Approach ii) and 9.89% of SNPs found unique to Approach iii).

The SNPs that are uniquely detected by Approach i) may represent SNPs that are present in a small subset of animals and hence are not representative of a specific RFI group. For SNPs with a low non-reference allele frequency, merging reads from multiple samples could lead to dilution of reads supporting the variant and consequently be called as homozygous for reference [32]. Alternatively, the Phred quality score of a SNP may be inflated when detected in a large number of samples and lead to some SNPs being uniquely detected by Approach i) (non-merged), which could have been removed by the quality filters in the merging methods suggesting possible false positives [33]. Alternatively, the detection of SNPs that are unique to the merging methods (Approach i) and Approach iii)) suggests that merging samples and tissues improves SNP detection and Phred quality scores due to the increased read depth and therefore reducing potential false positives.

### Comparison of RNA-Seq merging approaches based on whole transcriptome coverage, IGV visualization, and read depth coverage per variant

To determine the most optimized approach with highest read depth, the total reads mapped across the whole transcriptome for each approach were compared (Additional file 2). The analysis resulted in the total number of mapped reads on the reference for each individual sample in Approach i) (non-merged) and for merged map reads of samples in Approach ii) (merged by RFI group) and Approach iii) (merged by RFI and tissue group) (Additional file 2). On average, the total reads for Approach i) individual liver samples and individual muscle samples were 18,362,086.3 and 35,645,898.7, respectively. For Approach ii), the average total number of reads for each merged group of samples was 162,030,705, and for Approach iii) was 324,061,410. Approach iii) revealed the highest read depth and coverage across the whole transcriptome, suggesting that this approach may have higher read depth to filter out false positives and more accurately detect SNPs.

Average read depth coverage per variant was also determined. The descriptive statistics of the average read depth coverage per variant for each approach is shown in Table 3. For Approach i), read depth coverage per variant was 279.19 ± 2442.20 and 455.65 ± 3619.21, for liver and muscle respectively. For Approach ii), merged by RFI group, revealed an average read depth coverage per variant of 281.89 **±** 2457.76 and 461.60 **±** 3650.29, respectively. It is likely that muscle tissue displayed a higher read depth coverage per variant compared to liver, in both Approach i) and Approach ii), due to the higher number of reads for muscle tissue seen in (Additional file 1). For Approach iii) (merged by RFI and tissue group), an average read depth coverage per variant of 572.59 **±** 3993.11 was observed.

**Table 3:** Summary statistics for read coverage distribution per variant across approaches

Approach	Minimum	Median	Maximum	Mean ± SD	1st Quartile	3rd Quartile
Approach i)						
Liver	2	41	199,156	279.19 ± 2442.20	12	124
Muscle	2	55	199,974	455.65 ± 3619.21	13	218
Approach ii)						
Liver	2	41	199,156	281.89 ± 2457.76	12	125
Muscle	2	56	199,974	461.60 ± 3650.29	13	221
Approach iii)	2	62	209,060	572.59 ± 3993.11	13	280

Approach i) non-merged samples; Approach ii) merged samples by group for low-RFI and merged samples for high-RFI for each tissue, Approach iii) merged samples by group and tissue for low- and high-RFI for both tissues

*SD* Standard Deviation

**Fig. 1:**
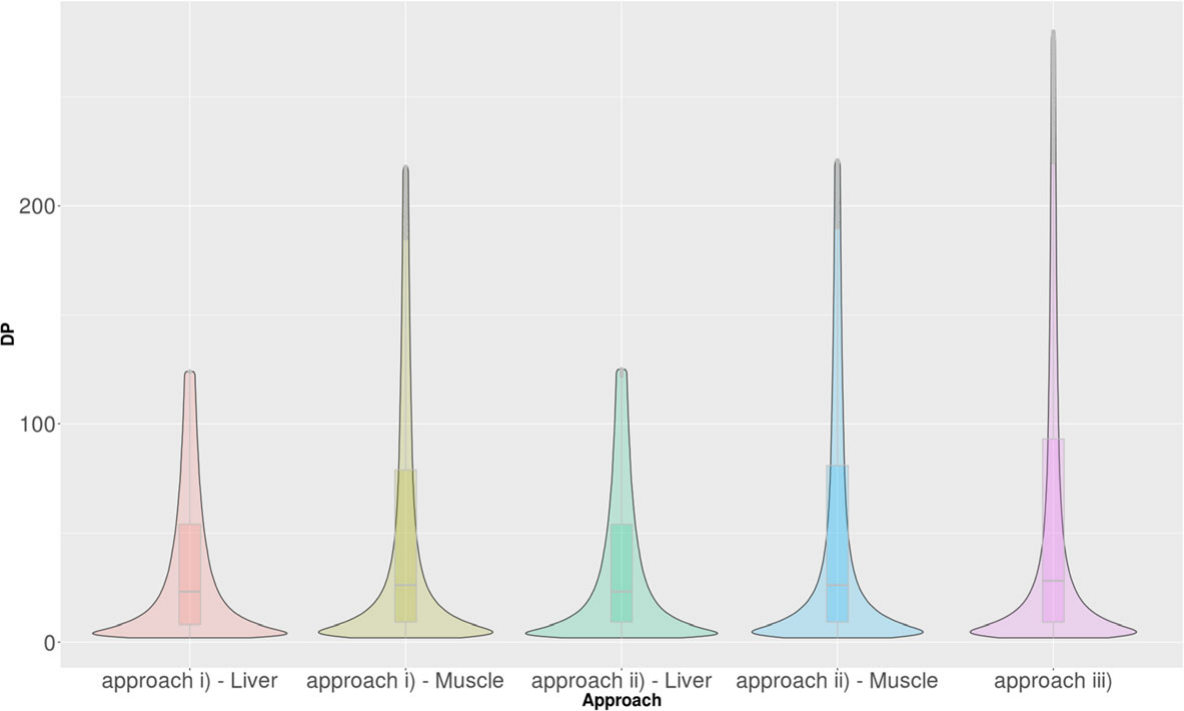
Violin plot of read coverage distribution of the variants detected in each approach. The plot is truncated after the 3rd quartile of the original read coverage distribution from each sample in order to improve the visualization due to the large number of observations distributed over a wide range. DP: Read depth per variant position for the corresponding approach. Approach i) non-merged samples; Approach ii) merged samples by group for low-RFI and merged samples for high-RFI for each tissue, Approach iii) merged samples by group and tissue for low- and high-RFI for both tissues.

The read depth coverage distribution, for the detected variants, for each approach is shown in Fig. 1. Approach iii) revealed the highest read depth per variant coverage, and the corresponding box plot showing the largest range between the 1st and 3rd quartile compared to the other approaches, indicates the high coverage for the detected variants. Furthermore, the plot also suggests that all the other approaches have a larger density of variants in the low coverage area; this is observed by the width of the box plot in each approach.

**Fig. 2:**
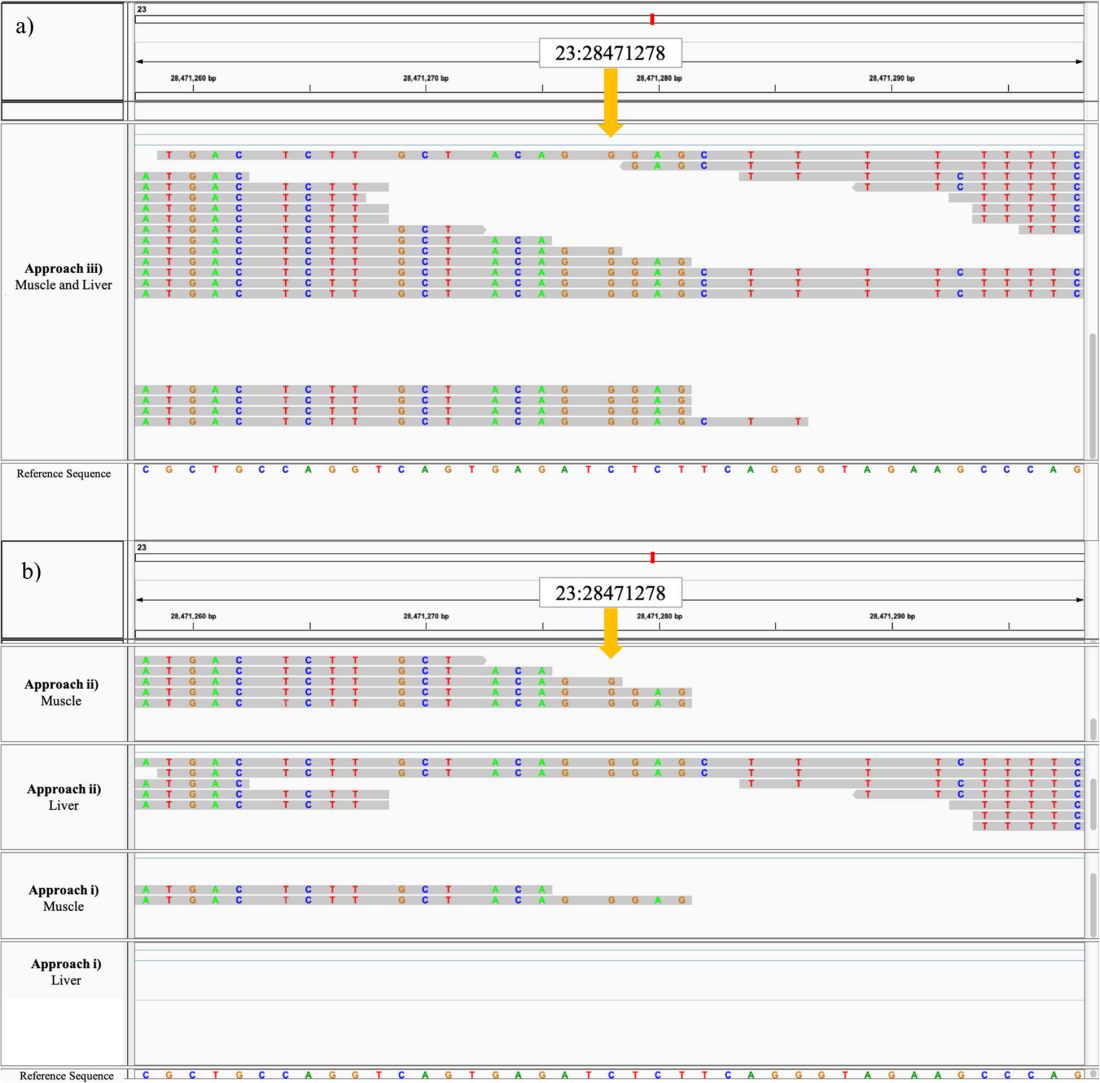
Visualization of the detection of an example variant (23: 28471278) using Approach iii), which is not detected by Approach i) or Approach ii), and corresponding read mapping. **a** Read mapping at example detected variant using Approach iii) Merged by RFI and tissue group; **b** Read mapping at example detected variant using Approach i) non-merged, and Approach ii) Merged by RFI group. Approach iii) Muscle and Liver: muscle and liver samples merged for low RFI .bam file. Approach ii) Muscle: merged muscle samples for low-RFI .bam file Approach ii) Liver: merged liver samples for low-RFI .bam file. Approach i) Muscle – non-merged individual muscle sample .bam file (sample accession number: ERS1342445). Approach i) Liver – non-merged individual liver sample .bam file (sample accession number: ERS579394). Approach descriptions: i) non-merged samples; ii) merged samples for low-RFI and merged samples for high-RFI for each tissue; iii) merged samples for low- and high-RFI for both tissues. Legend: Top numerical row (bp) = base pair position along transcriptome; bottom coloured row (bp letter) = UMD3.1 bovine reference genome (release 94) sequence. Coloured letters: Grey space = nucleotide base matches the reference base, Green = nucleotide base A, Red = nucleotide base T, Blue = nucleotide base C, Orange = nucleotide base G. Sequence region: Exon. Yellow arrow: Example variant at 23:28471278 detected by Approach iii) and not detected by Approach i) or Approach ii). Total read count coverage at variant site: Approach iii) = 10 (alternative allele = G (10), reference allele = C (0)); Approach ii) Muscle = 3 (alternative allele = G (3), reference allele = C (0)); Approach ii) Liver = 2 (alternative allele = G (2), reference allele = C (0)); Approach i) Muscle = 1 (alternative allele = G (1), reference allele = C (0)); Approach i) Liver = 0 (alternative allele = G (0), reference allele = C (0))

The increasing read depth and coverage across each approach can be visualized in Fig. 2 and (Additional file 4). As more samples are merged in Approach ii) and Approach iii), there is an increase in read depth, with Approach iii) displaying the greatest read depth. Similarly, when observing read depth coverage in [Additional file 4], read depth coverage increases as more samples are merged. Figure 2 displays the detection of a variant (chr:position; 23:28471278) in the low-RFI group using Approach iii) due to the increased read depth of 10, which is not detected in Approach i) or Approach ii) due to the lower read depth of 10. It is important to note that when increasing read depth by merging samples, the increase in read depth is not accumulative to the exact reads per .bam file. This is because after merging samples, read depth increases, but filtering processes for quality influences which reads are kept for variant calling based on the sequence quality (which is expected to increase when merging samples). This is the reason that we do not observe an exact sum of reads from .bam files in Approach iii) (Fig. 2. a) and b)).

This further supports the results from the whole transcriptome analysis, suggesting the increased read depth coverage across the whole transcriptome (Additional file 2), as well as the increased read depth coverage per variant (Table 3, Fig. 1), which is increased as we merge more samples across each approach. These results show that Approach iii) (merged by RFI and tissue group) has the highest read depth coverage across the whole transcriptome as well as the highest read depth coverage per variant, indicating the improved variant calling due to increased read depth.

### Comparison of quality of detected variants by each approach

As displayed in (Additional file 3), Cohen’s *d* values for Welch test illustrate the comparison of effect sizes of variant quality (QUAL) (defined as the Phred-scaled probability that a reference/alternative polymorphism exists at that site, based on the sequencing data), per detected variant between approaches. The Cohen’s *d* test suggests a large effect is ≥0.50 (Cohen, 1998), but may vary across disciplines.

When observing the Cohen’s *d* values (Additional file 3), the lowest values are observed when comparing the different tissues within the same approach (i.e., Approach i) a) non-merged (liver) and b) non-merged (muscle) = 0.035; Approach ii) a) merged by RFI group (liver) and b) merged by RFI group (muscle) = 0.020). This result is reasonable as it is expected that the coverage of reads of two tissues from individual samples would be similar (with variation in the genes/mRNA reads being expressed by each tissue), and therefore lead to similar variant calling quality. Similarly, the effect value when comparing the coverage of Approach ii) a) merged by RFI group (liver) and b) merged by RFI group (muscle) was also low (0.020), supporting this hypothesis (Additional file 3).

Low values of 0.015 and 0.034 were also observed when comparing Approach ii) a) merged by RFI group (liver) with Approach iii) merged by RFI and tissue, and Approach ii) b) merged by RFI group (muscle) with Approach iii) merged by RFI and tissue, respectively (Additional file 3). This may suggest that when merging by RFI group (Approach ii), the quality of detected variants may be similar to the quality of detected variants when merging by RFI group and tissue (Approach iii). This may be due to the higher coverage seen in Approach iii), illustrated in Fig. 1. This is further supported by the Cohen’s *d* value when comparing Approach ii) and Approach iii) (0.151), which is much lower than the comparison between Approach i) v.s. Approach ii) (0.554), and Approach i) v.s. Approach iii) (0.457), which are expected to have much larger difference in coverage (read depth) due to the merging of samples (Additional file 3), leading to improved variant calling quality. This is supported by the reported total reads mapped across transcriptome [Additional file 2] and reported coverage in Fig. 1, where total reads mapped and coverage are much higher in merged approaches (Approach ii) and iii)) compared to Approach i) (non-merged). The results reported show the differences in variant calling quality that further support Approach iii) which has demonstrated the highest coverage (Fig. 1) and read depth (Additional file 2).

**Fig. 3:**
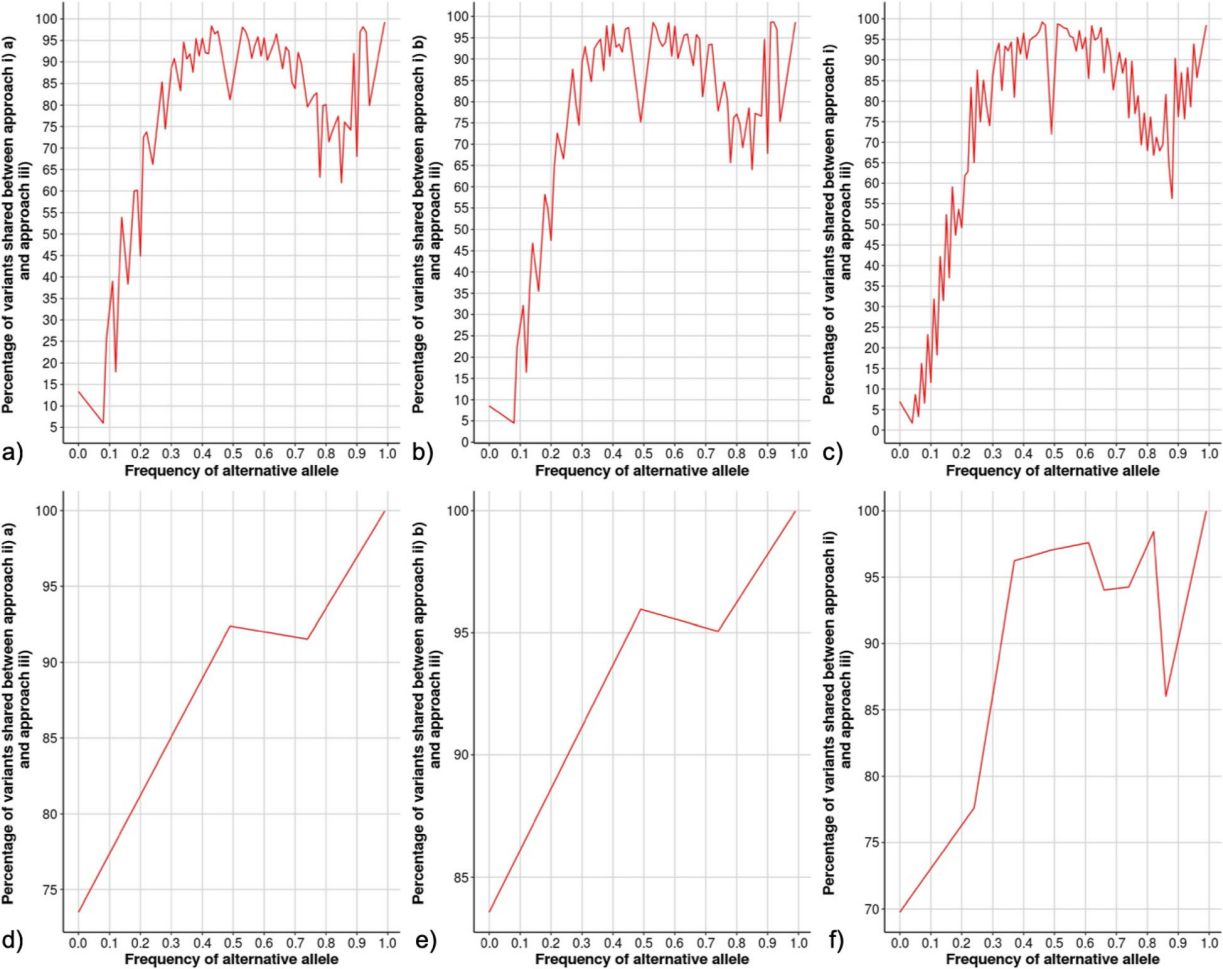
Alternative allele frequency by percentage of shared variants between two approaches. **a** Approach i) a) and Approach iii); **b)** Approach i) b) and Approach iii); **c)** Approach i) and Approach iii); **d)** Approach ii) a) and Approach iii); **e)** Approach ii) b) and Approach iii); **f)** Approach ii) and Approach iii). Approach i) = Non-merged samples (liver and muscle tissue); Approach i) a) = Non-merged samples (liver); Approach i) b) = Non-merged samples (muscle); Approach ii) = Merged samples for low-RFI and merged samples for high-RFI for each tissue (liver and muscle tissue); Approach ii) a) = Merged samples for low-RFI and merged samples for high-RFI for each tissue (liver tissue); Approach ii) b) = Merged samples for low-RFI and merged samples for high-RFI for each tissue (muscle tissue); Approach iii) = Merged samples for low- and high-RFI for both tissues (liver and muscle tissue)

Additional validation was performed to provide further evidence suggesting the most optimal approach by evaluating the proportion of variants detected by Approach i) and ii) against Approach iii), based on alternative allele frequency of the variants among the samples, which is illustrated in Fig. 3. A variant with low alternative allele frequency among samples means that the genotype of all samples at that detected variant site presents a low number of reads supporting this allele (non-reference/alternative alleles). This may suggest the variant was detected in a low number of animals (or small subset of animals), which are common in non-merged samples (Approach i)). Each comparison plot in Fig. 3 illustrates that the increase in samples with the alternative allele frequency (increase in samples with the detected variant/alternative allele), results in an increase or likelihood that they will be detected by both Approach i) or ii) and Approach iii). This indicates that variants with higher frequency of the alternative allele are more likely to be detected by both methods, and variants with low frequency of the alternative allele as less likely to be detected by both methods (Fig. 3). Therefore, variants with low frequency alternative allele may be non-representative of the population or considered as false positives when the objective is to detect candidate variants associated with a trait over a whole population or extreme phenotypic group.

It is also observed in each comparison (Fig. 3), that the detection of variants with alternative allele frequency reach a threshold of approximately 70% and begin to plateau; this may serve as the threshold in which regardless of adding additional samples, the alternative allele is detected by both approaches. Furthermore, it is important to highlight that when observing the plots illustrating detection of alleles based on alternative allele frequency between the merged sample approaches (Approach ii) merged by RFI group and Approach iii) merged by RFI and tissue group) (Fig. 3. d), e), and f))., the smallest percentage of shared variants is 70%, suggesting that several false positive variants are detected in the non-merged approach (Approach i)).

### Comparison of RNA-Seq merging approaches based on annotated SNPs, and annotated genes fixed within RFI groups

**Fig. 4:**
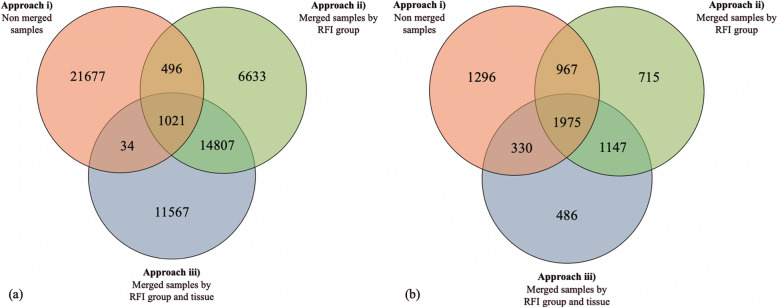
**a** Common and unique **SNPs** which were unique within low- or high-RFI groups across each approach. **b** Common and unique **genes** which were unique within low- or high-RFI groups across each approach. Approach i) = Non-merged samples Approach ii) = Merged samples for low-RFI and merged samples for high-RFI for each tissue. Approach iii) = Merged samples for low- and high-RFI for both tissues

**Fig. 5:**
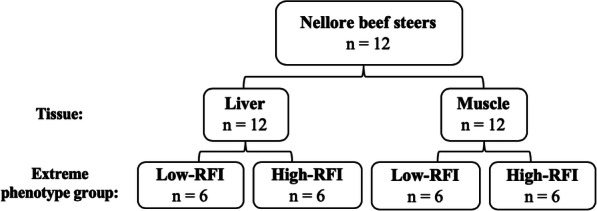
Population, tissue sample, and feed efficiency group structure using RNA-Seq data from two studies*. RFI = residual feed intake; *n* = sample size. Initial population sizes were *n* = 20 (liver) and *n* = 20 (muscle); however, 4 individuals were removed from each high-RFI group due to non-matching animal IDs. Additionally, 4 individuals were randomly removed from each low-RFI group to maintain a consistent sample size of *n* = 6 for each RFI group. *GEO Accession ID: PRJEB7696 and PRJEB15314 for liver and muscle tissue data, respectively

The list of SNPs fixed within low- or high-RFI groups and filtered for Moderate, Modifier, and High functional impact were compared across each approach (Fig. 3a). The most optimized approach for detecting SNPs that best represented the feed efficiency trait was determined by comparing the SNP detection results from each approach. From this, SNPs fixed within low- or high-RFI groups, filtered for moderate, modifier, or high functional impact, were compared across each approach (Fig. 4a), and the associated genes harboring these SNPs were also compared across approaches (Fig. 4b). The total amount of SNPs after filtering, detected by each approach were 23,228, 22,957, and 27,429 for Approach i), ii), and iii), respectively (Fig. 4a). A large overlap of SNPs (14,807) between Approach ii) and iii) was observed which is likely due to the merging of samples in these approaches. The total amount of genes unique to low- and high-RFI groups identified for each approach were 4568, 4804, and 3938 for Approach i), ii), and iii), respectively. A higher average of SNPs detected per gene was observed for Approach iii) (1.832 SNPs/gene; SD = 1.564), compared to Approach i) (1.775 SNPs/gene; SD = 1.499) and Approach ii) (1.773 SNPs/gene; SD = 1.537), as Approach iii) had the highest SNPs per annotated genes ratio (Fig. 5). This may suggest that Approach iii) reveals more SNPs are influencing fewer genes in regulating feed efficiency.

As shown in the Venn Diagram in Fig. 4b, the majority of genes (1975) from each approach were identified as shared across all three approaches. Additionally, 1296, 715, and 486 genes were identified as unique to Approach i), ii), and iii), respectively. The number of genes shared between Approach i) and ii) was 967, and between ii) and iii) was 1147, and between i) and iii) was 330. When considering the total genes identified in each approach, Approach iii) shares the most genes among both Approach i) and ii) (50%), and 8.37% with Approach i), and 29.13% with Approach ii). The genes shared among all three approaches were of most importance and interest, as they were the genes most representative of the trait, and Approach iii) had the most genes located in this mutual group. Additionally, Approach iii) had the lowest percent of genes unique to its own group (12.3%). This may suggest that Approach iii) is most representative of the unique genes associated with feed efficiency, and was able to identify more positional candidate genes (486) that are likely to play a role in regulating feed efficiency, while potentially excluding false positive SNPs that may have been annotated for untrue genes identified by the other approaches. Notably, Approach ii) and iii) had more shared variants (86.70%) compared to the other approach comparisons in Table 2, and Approach ii) and iii) also have the most shared annotated genes (Fig. 4b). Similarly, Approach i) and iii) had the least shared variants (Table 2) and the least shared annotated genes (Fig. 4b). This suggests that the comparison of unique and shared SNPs across approaches in Table 2 is representative or may be correlated with the annotated genes (for low- and high-RFI) compared between approaches in Fig. 4b.

### Unique SNPs fixed within low- or high-RFI groups and their associated candidate genes and metabolic pathways

Total number of unique or shared SNPs identified across low- and high-RFI groups using Approach iii) are shown in (Additional file 5). The VCF files including the identified variants fixed within low- or high-RFI groups using the most optimized approach is available in (Additional file 6) and (Additional file 7), respectively. These VCF files are publicly available on the European Variation Archive platform under the Project PRJEB37881 in the file ERZ1307564 (HIGH_FE_1) for variants unique to low-RFI group and in the file ERZ1307563 (LOW_FE_2) for variants unique to the high-RFI group. In total, 415,624 SNPs were detected, with 13,145 SNPs unique in low-RFI, and 14,663 SNPs unique in high-RFI. In addition, 387,816 SNPs were identified as shared across both low- and high-RFI groups. The unique SNPs were filtered for variant impact information including only Moderate, Modifier, or High impact. With variant impact ‘High’, defined as the variant resulting in high or disruptive impact in the protein that would lead to protein truncation, loss of function, or tissue nonsense, mediated delay; ‘Moderate’, meaning a non-disruptive variant that may change protein effectiveness; or ‘Modifier’, meaning variant affecting non-coding genes, where predictions are difficult or the impact is unknown [34]. Further investigation of the genes associated with the SNPs that may impact protein function were selected as they are likely to have a functional impact on metabolic pathways that play a role in regulating feed efficiency. The VeP results including the variants identified uniquely within low- or high-RFI groups, using Approach iii) is reported in [Additional file 8], which includes variant positional, functional consequence, and associated gene information. In total, 2886 and 3075 genes, in which SNPs were located within, were identified for the low- and high-RFI groups, respectively. Results showed that 111 and 3 biological pathways were significantly enriched for the low- and high-RFI group genes, respectively. The gene subgroups associated with the significantly enriched biological pathways for low- and high-RFI groups is reported in (Additional file 9). The three most significant pathways associated with each RFI group are displayed in Table 4. Low-RFI animals display a genetic architecture with fewer genes affecting more biological pathways, compared to high-RFI animals, explaining the large number of significantly enriched biological pathways (111) for low-RFI group, compared to the high-RFI group (3). Additionally, when observing the total unique and shared SNPs detected by each approach (Fig. 4a), and the genes in which they are located in (Fig. 4b), it is observed that many variants may be influencing the same genes, and consequently biological pathways, simultaneously, as there is a larger SNP/annotated gene ratio. Alternatively, it is possible the subset of genes associated with the significantly enriched biological pathways are more specific or functionally relevant to each other for the high-RFI group, which had only 3 significantly enriched biological pathways. Additionally, it is known that feed efficiency which is known for intense selective pressure in beef cattle, has shown signatures of selection differentiation among multiple breeds and populations [35]. Over time, the intensive selection for feed efficiency may have influenced the shift in genomic regions and how they influence the metabolic regulation in a more or less feed efficient animal. Therefore, alternatively, from strong selective pressure on feed efficiency, it is possible the cumulative number of variants being selected, are increasing in frequency. This may lead to more variants affecting the trait in the population, which may explain the larger number of variants in the low-RFI group, which are influencing more biological pathways. In addition, although feed efficiency is a polygenic trait, core genes may still be influencing the regulation of affecting feed efficiency. Therefore, directional selection feed efficiency would increase the allelic frequency of variants of possible core genes.

**Table 4:** Biological pathways significantly associated with genes in which SNPs from Low- and High-RFI were localized

Pathway Name	***p***-value	FDR^i^	Genes associated with SNPs detected	Function
Low-RFI^ii^				
Members of the BCR^a^ signaling	1.68 × 10^−6^	5.12 × 10^−3^	20	Immune
Oxytocin signaling	1.70 × 10^−5^	1.87 × 10^−2^	42	Reproduction and Metabolism
EPHA2^b^ forward signaling	2.72 × 10^−5^	1.87 × 10^−2^	10	Growth and Metabolism
High-RFI				
Members of the BCR signaling	1.21 × 10^−5^	1.52 × 10^−2^	19	Immune
B cell activation	1.313 × 10^−5^	1.52 × 10^−2^	22	Immune
Regulation of RAC1^c^ activity	1.48 × 10^−5^	1.52 × 10^−2^	17	Metabolism

^i^ *FDR* False Discovery Rate

^ii^ Top 3 pathways out of 111 significantly associated pathways

^a^ *BCR* B Cell Receptor

^b^ *EPHA2* Ephrin type-A receptor 2

^c^ *RAC1* Ras-related C3 botulinum toxin substrate 1

When using RNA-Seq technology to detect genetic variants [4], the variants are expected to be detected from mRNA reads, which are not necessarily differentially expressed or highly expressed in a specific tissue. The following positional candidate variants, and genes they are located within, are discussed in a means that the positional variants are unique or fixed within RFI groups and are located within or near the discussed genes that could be of interest to better understand feed efficiency, but not necessary in terms of the expression of the genes.

The three significant pathways associated with low-RFI (more feed efficient) cattle, included immune response, fertility, and metabolism pathways, including the BCR signaling pathway, oxytocin signaling pathway, and *EPHA2* forward pathway (Table 4). The BCR signaling pathway is associated with immune response and fertility; Olivieri et al. [36] previously identified candidate genes related to feed efficient Nellore beef cattle which were associated with immune system function, including *NLRP14* gene which was present in the low feed efficient group in this study, and *CACNG7* which was present in high feed efficient group in this study. The regulation of *NLRP14* has also been suggested to be associated with excessive accumulation of undifferentiated spermatogonia germ cells in cattle, implying its role in reproductive function [37]. It may be possible that these genes which were overlapping with variants unique to low- and high-RFI steers are being expressed. The *CACNG7* gene is associated with oxytocin signaling; this may explain the oxytocin enriched pathway in the current study which was associated with more feed efficient cattle, supporting a link between metabolic processes with feed efficiency. Oxytocin is a neuropeptide which has also been found to regulate brown adipocyte production, which functions for metabolic maintenance of temperature regulation (thermogenesis) and gluconeogenesis [38].

The *EPHA2* forward signaling was also associated with more feed efficient cattle in this study. The *EPHA2* is a gene known to be targeted by miR-26b, a microRNA (miRNA) in pituitary tissues of Zanbian cattle, and this miRNA is known to regulate cell proliferation, differentiation, apoptosis, and development [39]. In addition, *EPHA2* receptor is a key modulator for a wide variety of cellular functions, such as embryonic development, tissue boundary formation, central nervous system function, bone remodeling, vascular organization [40], suggesting its role in metabolic maintenance and regulation. This may be relevant in the regulation of feed efficiency due to the strong correlation between feed efficiency and efficiency of metabolic energy use to meet physiological demands.

The three significant pathways associated with high-RFI (less feed efficient) cattle, also included BCR signaling pathway (Table 4), which may suggest similar variants are present in both low- and high-RFI groups, but are acting in different ways to regulate feed efficiency in cattle. This is supported by the discovery in the study where many SNPs were detected in both low- and high-RFI groups with the same position but with different alleles. Another significantly associated pathway to less feed efficient cattle was B cell activation which is additionally related to immune response. It has been found that B cell activation pathway was enriched for differentially expressed genes in pigs selected for RFI [41]. This may suggest the influence of feed efficiency on immune pathways and function in livestock. Regulation of *RAC1* was another significantly associated pathways with less feed efficient cattle, which is known to have a role in glucose transport and skeletal muscle [42], and could be associated with the regulation of gluconeogenesis, along with oxytocin as discussed previously.

Overall, the most significant pathways associated with the extreme feed efficiency groups were both related to metabolic, reproductive, and immune functions (Table 4). Notably, both feed efficiency groups found similar significant pathways. From the analysis, similar SNP were found with different alleles in both low- and high-RFI, suggesting that the same SNPs with different alleles are present in animals in both high and low feed efficiency groups, influencing genes differently, to make an animal more or less feed efficient.

Further studies using orthogonal strategies such as Sanger sequencing, target sequencing, and whole genome sequencing as truth sets could serve as further evidence to confirm the optimized approaches (by validating variant calling sensitivity and specificity for variants), as well as the functional positional variants associated with a specific trait.

## Conclusions

In conclusion, this study demonstrates the different results obtained in SNP detection from using different sample merging pipelines for RNA-Seq analysis. We suggest that the present optimized pipeline for SNP detection when analyzing multiple samples and tissues in divergent traits or phenotypic groups is to merge multiple sample and tissue data by group to increase aligned reads at each variant site which increases whole transcriptome read depth coverage, as well as read depth coverage per variant, and variant calling quality, leading to improved accuracy and power of SNP detection and reduction of false positive detection. This pipeline can be used to identify SNPs associated with extreme phenotypes of interest, which can be annotated to identify genes that may be important in the regulation of the trait. Overall, this work presents an optimized RNA-Seq pipeline to discover SNPs in coding regions to improve the detection of potential trait-associated variations using RNA-Seq data. Additionally, from using the proposed optimized RNA-Seq pipeline, this study successfully identified functional candidate SNPs within genes involved in major biological pathways associated with high and low feed efficient Nellore beef cattle. This suggests the relationship between immune, reproductive, and metabolic function with feed efficiency in beef cattle, and confirming the successful use of the most optimized RNA-Seq pipeline described in this study.

## Methods

### RNA-Seq dataset

The datasets used in this study consist of RNA-Seq data obtained from liver and muscle tissue samples from Nellore beef steers deposited in the NCBI - Gene Expression Omnibus (GEO) public repository with PRJEB7696 and PRJEB15314 GEO accession numbers for both liver and muscle, respectively. Detailed animal management and sampling information was previously described by Tizioto et al. [43] and by Tizioto et al. [44]. Briefly, 20 Nellore feedlot beef steers (*n* = 10 low-RFI and *n* = 10 high-RFI) at 21 months of age were used, which were selected from 585 steers that were calculated for feed efficiency through Best Linear Unbiased Prediction (BLUP) estimates for RFI [43, 44]. Tissue samples were collected from the longissimus thoracic muscle [44] and liver [43]. RNA was extracted using Trizol method (Invitrogen®), and mRNA sample preparation was performed using TruSeq RNA Sample Preparation Kit® (illumina, San Diego, CA). Cluster generation and sequencing was performed on the Illumina HiSeq 2000® which generated paired end-reads of 2 × 100 bp. The previous studies performed identification and annotation of differentially expressed genes between feed efficiency groups [43, 44].

Comparison of animals from each study revealed four high-RFI individuals in the muscle group which did not match the high-RFI individuals in the liver group. This may be due to RFI re-ranking of animals, as liver tissue was sampled from only 83 of the 585 beef steers. Therefore, the four non-matching animals from the high-RFI muscle group (ERS1342436, ERS1342439, ERS1342440, ERS1342443) were removed from the study, and four corresponding individuals (ERS579404, ERS579406, ERS579407, ERS579411) from the high-RFI liver group were also removed. To maintain the same sample size across RFI groups, four animals were randomly selected to be removed from the low-RFI group for muscle (ERS1342447, ERS1342448, ERS1342450, ERS1342453) and liver (ERS579395, ERS579396, ERS579398, ERS579301). Therefore, this study used RNA-Seq data from 12 Nellore beef steers divergent for feed efficiency (*n* = 6 low-RFI and *n* = 6 high-RFI) from both muscle and liver tissue (Fig. 5). The sample size of 6 animals per extreme phenotypic group is sufficient and in the optimal range for RNA-Seq studies as described by Soneson and Delorenzi [45].

### RNA-Seq analysis workflow

**Fig. 6:**
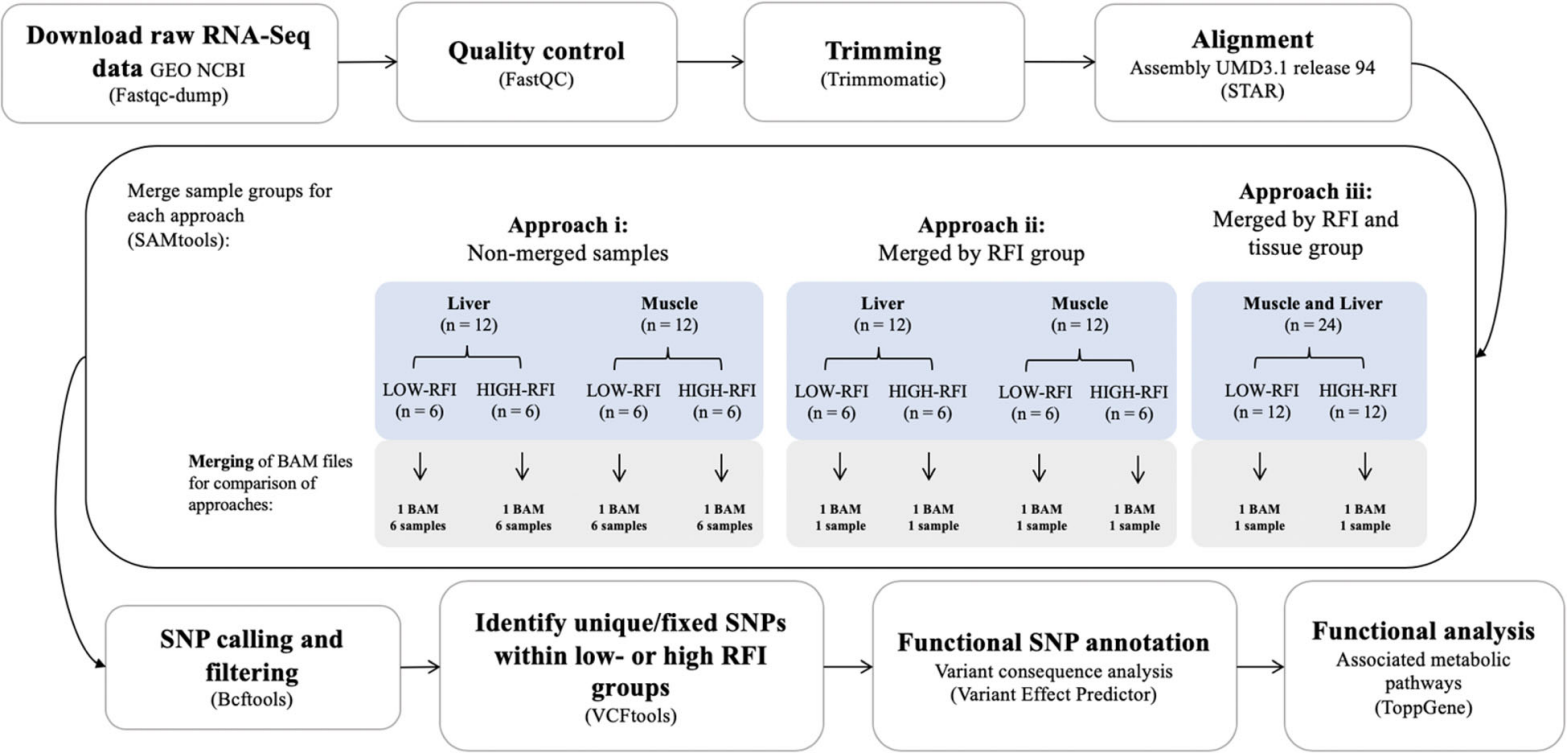
Workflow diagram to compare SNP calling approaches, identify functional SNP, and predict corresponding variant effects. ^i^ Samples were merged based on three different approaches: i) Non-merged samples, meaning BAM files were not merged and each file was called individually using a multi-sample VCF file containing 6 GT (1 for each animal) for each group; ii) Merged samples for low-RFI and merged samples for high-RFI for each tissue, meaning BAM files of same group and tissue were merged. Using one VCF file for each RFI group per tissue, containing 1 GT; iii) Merged samples for low- and high-RFI for both tissues, meaning BAM files of same group were merged for tissues. Using one VCF file for each RFI group for both tissues, containing 1 GT. RFI = residual feed intake; GT = genotype; SNP = single nucleotide polymorphism; VCF = variant calling format

Identification of SNPs was performed using the workflow shown in Fig. 6, and the complete RNA-Seq pipeline script can be seen in [Additional file 10]. Fastq files were downloaded using the SRA toolkit command fastq-dump with the option --split-files to download the data from each sample into two files, one for each of the paired ends. Quality of sequence reads were verified using FastQC (version 0.11.8 [46];) to identify sequencing read artifacts including sites with low quality Phred scores, duplicated reads, uncalled bases (N sequences), and potential contamination [47, 48]. Next, reads were trimmed to remove Illumina adapters and low quality bases at the start and end of reads (sites removed if Phred score < 30) using Trimmomatic (Version 0.38 [49];). Additionally, reads with an average quality score below 20 within a sliding window of 5 nucleotides, and with length less than 75 bp were removed. Quality of sequence reads were re-evaluated post-trimming using FastQC [46]. The resulting trimmed reads from each sample were individually aligned to the bovine reference genome (*Bos Taurus* Assembly UMD3.1. release 94), using STAR (version 2.7.0 [50];). with the following filtering options: --outFilterMismatchNmax 999, allowing a maximum of 999 mismatches per pair, −-outFilterMismatchNoverReadLmax 0.04, allowing alignment to be output only if its ratio of mismatches to read length is less than 0.04, and --outFilterMultimapNmax 1, which allowed a max of 1 alignment per read (if exceeded, the read is considered unmapped). Use of the updated ARS-UCD 2.1 bovine assembly [51] may reveal more efficient and accurate assembly due to improved sequence quality and annotation [52]. However, assembly improvements are more likely to improve annotation of the exons of positional and functional candidate genes, as opposed to genetic variants such as SNPs and INDELs. It is expected that use of an updated assembly would lead to similar results. Following alignment, ReadGroups (RG) were then added to each sample and PCR duplicates were marked and removed using PICARD tools (Version 2.18.25.; http://broadinstitute.github.io/picard/). The RGs allowed for differentiation of samples by assigning the origin of the read (low- or high-RFI group) and assignment of SNPs to a specific genotype.

### RNA-Seq read merging approaches

A diagram outlining the strategy used for merging BAM files is shown in (Additional file 11). Three different RNA-Seq read merging approaches were compared including: i) non-merged samples, ii) merged samples by group for low-RFI and for high-RFI for each tissue, and iii) merged samples by group and tissue for low- and high-RFI for both tissues (liver and muscle). Each approach required the merging of aligned reads prior to variant calling (Fig. 6). BAM files were merged using SAMtools option ‘samtools merge’ (Version 1.4 [53];), which merged multiple BAM files and produced a single output file per strategy, leading to a total of six new BAM files containing merged aligned reads from: low-RFI muscle samples, low-RFI liver samples, high-RFI muscle samples, high-RFI liver samples, low-RFI muscle and liver samples, and high-RFI muscle and liver samples [Additional file 11].

### Variant calling and filtering

Variant calling was performed for each read merging method to identify SNPs using the ‘mpileup’ and ‘call’ commands from BCFtools (Version 1.9–77-gd0cf724+ [54];). This involved conversion of BAM files into genomic positions and variant calling, producing a BCF (Binary Variant Call Format) file containing variant information including genomic position, alternative allele detected, quality of SNP call, and other information (Danacek et al., 2011). The multi-sample calling (or joint calling) method, previously shown to enhance the sensitivity of SNP detection and accuracy of genotype calling over calling each sample independently in datasets with low sequencing depth, was employed for all read merging approaches [19, 25].

The comparisons of approaches performed with VCF files of varying genotypes (GT) is summarized in [Additional file 11]. For Approach i), BAM files (aligned reads) from the different animals and tissues were not merged and files for each tissue type were called for variants individually using multi-sample calling, resulting in one multi-sample VCF file for each tissue type, each containing 12 genotypes (1 GT for each animal). For Approach ii), BAM files of animals in the same RFI group were merged for each tissue and files for each tissue type were called for variants using multi-sample calling, resulting in one VCF file for each tissue, each containing 2 genotypes (1 GT per RFI group). For Approach iii), BAM files of animals in the same RFI group were merged for both tissues prior to multi-sample calling, resulting one VCF file that contained 2 genotypes (1 GT per RFI group). Variant filtering was performed using VCFtools to remove variants with a minimum read depth below 10 and a minimum of 2 supporting reads for the alternative allele as well as to filter SNPs within 3 bp surrounding a gap as described by Cánovas et al. [4]. BCFtools filter was used to remove variants with quality values below 30 (based on Phred scaled scores for the assertion made in the alternative allele), filter SNPs within 5 bp of an INDEL, and filter any alternative allele with a lower frequency of 20% in the population. All analyses described regarding the RNA-Seq pipeline [Additional file 10] was performed using shell processor with 6 cores, 1600 mgH processing capacity, and total RAM memory of approximately 100 GB.

### Whole Transcriptome coverage, read depth coverage per variant, and IGV visualization analysis

The Whole Transcriptome Coverage Analysis tool of CLC Genomics Workbench 12.0.2 (https://www.qiagenbioinformatics.com/) was used to determine the total number of unfiltered reads across the whole transcriptome or each individual sample in Approach i) and for merged sample by groups and tissues in Approach ii) and iii). This tool is used to identify regions in read mappings with unexpectedly low or high coverage, however, the summary of results of this analysis presents the ‘Total Mapped Reads’ across the whole transcriptome for each approach, counting both reads of a paired end sequence, while ignoring ‘non-specific matches’, or ‘broken pairs’ (CLC Genomics Workbench 12.0.2, https://www.qiagenbioinformatics.com/).

To further evaluate the read coverage across the approaches, the read coverage (DP) per variant distribution for each approach was calculated. The read depth per variant for each approach was plotted (Fig. 1) and corresponding summary statistics were reported (Table 3). The statistical summary of the read depth per variant coverage includes the minimum, maximum, median, read depth at the 1st and 3rd quartile, and standard deviation of the read depth coverage per variant for each approach (Table 3).

Integrative Genomics Viewer (IGV; https://software.broadinstitute.org/software/igv/) was used to visualize the read mapping and coverage (depth of the reads displayed at each locus) between each approach. As an example, the IGV visualizations illustrates the read mapping (Fig. 2), and coverage [Additional file 4], at a randomly selected variant which was detected exclusively in Approach iii) (chr:position; 23:28471278) in the low-RFI group. The IGV visualizations include the .bam file of Approach iii), Approach ii) liver, Approach ii) muscle, and one example sample each from Approach i) muscle and Approach i) liver. This allowed for an illustrated example of how read depth (Fig. 2) and coverage (Additional file 4) increases as we merge more samples across each approach.

### Comparison of approach variant quality and coverage analysis

Further evaluation of the suggested most optimized approach (Approach iii); merged samples by RFI and tissue group) was performed by calculating the Cohen’s *d* value for Welch test (Cohen 1988), which compared the effect sizes of the quality (QUAL) in VCF files of detected variants in each approach. Quality (QUAL) value in VCF files represent the Phred-scaled probability that a reference/alternative polymorphism exists at the variant site, based on the sequencing data. Cohen’s *d* value was calculated using the lsr package in R assuming non equal variances between groups (R Version 3.6.0 [55];). All Cohen’s *d* values for each approach comparison are reported in (Additional file 3).

Additionally, the stats and plot-vcfstats options from bcftools software [54] were used to evaluate the proportion of detected variants based on alternative allele frequency between Approach i) a) non-merged (liver) and b) non-merged (muscle) and Approach ii) a) merged by RFI group (liver) and b) merged by RFI group (muscle), against Approach iii) merged samples by RFI and tissue group (Fig. 3). Plots were developed based on custom R script (R Version 3.6.0 [55];).

### Identification and gene annotation of unique SNPs fixed within low- or high-RFI groups for each approach

Unique SNPs fixed within low- or high-RFI groups were identified using the VCF files containing filtered SNPs as previously described for each approach. These SNPs were filtered for moderate or high functional impact using Variant Effect Predictor (VeP) [34]. Variants with functional consequence within High, Moderate, or Modifier categories were selected for further analysis. In addition, in order to identify positional candidate genes, the genes localized with these SNPs were also determined using custom R scripts (R Version 3.6.0 [55];). and the VennDiagram R package [13, 47].

Approach iii) was then used to identify unique SNPs fixed within low- or high-RFI groups using the VCF files containing filtered SNPs as previously described (Fig. 6). The VCF files containing the uniquely detected variants within low- or high-RFI groups using Approach iii) is reported in (Additional file 6( and (Additional file 7) respectively. In addition, the VCF files are publicly available on the European Variation Archive platform under the Project PRJEB37881 in the file ERZ1307564 (HIGH_FE_1) for variants unique to low-RFI group and in the file ERZ1307563 (LOW_FE_2) for variants unique to the high-RFI group. The 2 VCF files were then compared (1 low-RFI and 1 high-RFI VCF) with 1 GT each (representative of multiple samples). SnpSift (Version 4.0 [56];) filtering command was used to remove all variants present in one RFI group but missing in the other RFI group. The VCF file was then split using VCFtools vcf-subset to create one VCF file with only low-RFI variants and one VCF file with only high-RFI variants, which were then compared using the BCFtools isec command to determine the intersection of the files and create three files: SNPs exclusive to low-RFI VCF file, SNPs exclusive to high-RFI VCF file, and SNPs shared between both low- and high-RFI groups VCF file. Positional candidate genes in which these variants were localized were selected to determine associated metabolic pathways. Functional annotation was performed using ToppGene [57] to determine metabolic pathways significantly associated with the gene lists. Briefly, the annotated genes of the detected SNPs fixed within the low- or high-RFI group using Approach iii) were input as a low-RFI gene list and high-RFI gene list using ‘ToppFun’ function for gene list functional enrichment analysis. This function uses hypergeometric distribution with Bonferroni correction and displays statistically significant results (FDR < 0.05) for multiple annotation categories based on the gene list input. This study focused on the annotation category ‘Biological Pathways’.

## Supplementary information

**Additional file 1.**

**Additional file 2.**

**Additional file 3.**

**Additional file 4.**

**Additional file 5.**

**Additional file 6.**

**Additional file 7.**

**Additional file 8.**

**Additional file 9.**

**Additional file 10.**

**Additional file 11.**

## Data Availability

All data generated or analysed during this study are included in this published article [and its supplementary Additional files]. The datasets analyzed during the current study include RNA-Seq datasets which are available in the NCBI - Gene Expression Omnibus (GEO) public repository under [https://www.ncbi.nlm.nih.gov/bioproject/PRJEB7696/] and [https://www.ncbi.nlm.nih.gov/bioproject/?term=PRJEB15314] GEO accession numbers for liver (PRJEB7696) and muscle (PRJEB15314) tissue, respectively. The data of the variants uniquely detected in low- or high-RFI groups using Approach iii) are available in the European Variation Archive (https://www.ebi.ac.uk/eva/?Home), archived under project PRJEB37881 in the submission file names ERZ1307563 (LOW_FE_2) and ERZ1307564 (HIGH_FE_1), which are reported in this study as (Additional file 6) and (Additional file 7), respectively.

## Abbreviations

BAM: Binary alignment map
BCF: Binary calling format
BCR signaling pathway: B cell antigen receptor signalling pathway
BLUP: Best linear unbiased prediction
bp: Base pair
CACNG7: Calcium voltage-gated channel auxiliary subunit gamma 7
chr: Chromosome
DP: Read coverage
EPHA2: Ephrin type-A receptor 2
eQTL: Expression quantitative trait loci
FAO: Food and agriculture organization of the United Nations
FDR: False discovery rate
FE: Feed efficiency
GATK: Genomic analysis toolkit
GEO: Gene expression omnibus
GT: Genotype
High-RFI: High-residual feed intake (low feed efficient)
IGV: Integrative genome viewer
Low-RFI: Low-residual feed intake (high feed efficient)
miRNA: MicroRNA
NLRP14: NLR family pyrin domain containing 14
QTL: Quantitative trait loci
RAC1: Rac family small GTPase 1
RFI: Residual feed intake
RNA: Ribonucleic acid
RNA-Seq: RNA-sequencing
SD: Standard deviation
SNP: Single nucleotide polymorphism
VCF: Variant calling format
VeP: Variant effect predictor
