# Supplementary material for: Development and comparison of RNA-sequencing pipelines for more accurate SNP identification: practical example of functional SNP detection associated with feed efficiency in Nellore beef cattle
Source: BMC Genomics. 2020 Oct 8;21:703. doi: 10.1186/s12864-020-07107-7 (PMC7545862; doi:10.1186/s12864-020-07107-7)
Supplement: Supplementary file 1 — Additional file 1. [file 12864_2020_7107_MOESM1_ESM.docx]

**Additional file 1.** Liver and muscle sample information including feed efficiency group and read mapping information.

| **Sample Accession Number by study** | **Feed efficiency group** | **Total Reads** | **# uniquely mapped reads** | **% uniquely mapped reads** |
| --- | --- | --- | --- | --- |
| Tissue (study accession #) | | | | |
| Liver (PRJEB7696) | | | | |
| ERS579394 | high | 7,338,446 | 6,975,563 | 95.06 |
| ERS579397 | high | 7,079,855 | 6,714,099 | 94.83 |
| ERS579399 | high | 8,325,791 | 7,903,077 | 94.92 |
| ERS579400 | high | 7,716,404 | 7,305,248 | 94.67 |
| ERS579402 | high | 7,354,529 | 6,978,182 | 94.88 |
| ERS579403 | high | 7,114,744 | 6,742,752 | 94.77 |
| ERS579405 | low | 6,363,266 | 6,042,120 | 94.95 |
| ERS579408 | low | 6,596,129 | 6,272,955 | 95.10 |
| ERS579409 | low | 7,923,682 | 7,497,828 | 94.63 |
| ERS579410 | low | 7,132,356 | 6,642,434 | 93.13 |
| ERS579412 | low | 6,913,752 | 6,537,053 | 94.55 |
| ERS579413 | low | 7,607,639 | 7,209,026 | 94.76 |
| Muscle (PRJEB15314) | | | | |
| ERS1342445 | high | 17,227,810 | 16,261,921 | 94.39 |
| ERS1342446 | high | 20,976,425 | 19,931,762 | 95.02 |
| ERS1342449 | high | 20,926,000 | 17,475,676 | 83.51 |
| ERS1342451 | high | 14,807,990 | 14,064,136 | 94.98 |
| ERS1342452 | high | 15,773,643 | 10,430,495 | 66.13 |
| ERS1342454 | high | 13,852,120 | 13,174,573 | 95.11 |
| ERS1342435 | low | 18,443,978 | 17,618,969 | 95.53 |
| ERS1342437 | low | 15,475,954 | 14,733,896 | 95.21 |
| ERS1342438 | low | 17,516,264 | 16,728,409 | 95.50 |
| ERS1342441 | low | 17,307,502 | 16,462,170 | 95.12 |
| ERS1342442 | low | 14,900,733 | 14,179,347 | 95.16 |
| ERS1342444 | low | 18,061,449 | 17,254,366 | 95.53 |
