# Supplementary material for: Development and comparison of RNA-sequencing pipelines for more accurate SNP identification: practical example of functional SNP detection associated with feed efficiency in Nellore beef cattle
Source: BMC Genomics. 2020 Oct 8;21:703. doi: 10.1186/s12864-020-07107-7 (PMC7545862; doi:10.1186/s12864-020-07107-7)
Supplement: Supplementary file 2 — Additional file 2. [file 12864_2020_7107_MOESM2_ESM.docx]

**Additional file 2.** Mapped total reads across transcriptome for each approach.

| **Sample Accession number or samples merged description** | **Feed efficiency group** | **Mapped Total Reads** |
| --- | --- | --- |
| **Approach i): Non-merged** |  |  |
| **Individual liver samples** |  |  |
| ERS579394 | high | 18,505,235 |
| ERS579397 | high | 17,915,515 |
| ERS579399 | high | 21,085,175 |
| ERS579400 | high | 19,602,337 |
| ERS579402 | high | 18,583,880 |
| ERS579403 | high | 18,078,573 |
| ERS579412 | low | 17,489,472 |
| ERS579413 | low | 19,044,144 |
| ERS579408 | low | 16,647,703 |
| ERS579409 | low | 19,843,569 |
| ERS579410 | low | 17,775,528 |
| ERS579405 | low | 15,773,905 |
| **Individual muscle samples** |  |  |
| ERS1342445 | high | 35,720,847 |
| ERS1342446 | high | 43,782,111 |
| ERS1342449 | high | 43,403,981 |
| ERS1342451 | high | 30,832,942 |
| ERS1342452 | high | 33,478,392 |
| ERS1342454 | high | 28,850,693 |
| ERS1342435 | low | 38,307,806 |
| ERS1342437 | low | 32,252,078 |
| ERS1342438 | low | 36,486,302 |
| ERS1342441 | low | 36,091,768 |
| ERS1342442 | low | 31,067,715 |
| ERS1342444 | low | 37,476,149 |
| **Approach ii): Merged by RFI group** |  |  |
| Liver samples for high feed efficiency merged | high | 113,770,715 |
| Liver samples for low feed efficiency merged | low | 106,601,321 |
| Muscle samples for high feed efficiency merged | high | 216,068,966 |
| Muscle samples for low feed efficiency merged | low | 211,681,818 |
| **Approach iii): Merged by RFI group and tissue group** |  |  |
| Liver and Muscle samples for high feed efficiency merged | high | 329,839,681 |
| Liver and Muscle samples for low feed efficiency merged | low | 318,283,139 |

*Mapped total reads - refers to total reads based on coverage across transcriptome
