# Supplementary material for: Development and comparison of RNA-sequencing pipelines for more accurate SNP identification: practical example of functional SNP detection associated with feed efficiency in Nellore beef cattle
Source: BMC Genomics. 2020 Oct 8;21:703. doi: 10.1186/s12864-020-07107-7 (PMC7545862; doi:10.1186/s12864-020-07107-7)
Supplement: Supplementary file 3 — Additional file 3. [file 12864_2020_7107_MOESM3_ESM.docx]

| **Approach comparison** | **Cohen’s *d* value** |
| --- | --- |
| Tissue comparison within same approach | |
| Approach i) a) v.s. Approach i) b) | 0.035 |
| Approach ii) a) v.s. Approach ii) b) | 0.020 |
|  |  |
| Approach comparison within same tissue | |
| Approach i) a) v.s Approach ii) a) | 0.624 |
| Approach i) b) v.s. Approach ii) b) | 0.625 |
|  |  |
| Approach i) tissues comparison with other Approaches | |
| Approach i) a) v.s. Approach i) | 0.155 |
| Approach i) a) v.s. Approach ii) | 0.718 |
| Approach i) a) v.s. Approach iii) | 0.625 |
| Approach i) b) v.s. Approach i) | 0.187 |
| Approach i) b) v.s. Approach ii) | 0.731 |
| Approach i) b) v.s. Approach iii) | 0.642 |
|  |  |
| Approach ii) tissues comparison with other Approaches | |
| Approach ii) a) v.s. Approach i) | 0.454 |
| Approach ii) a) v.s. Approach ii) | 0.173 |
| Approach ii) a) v.s. Approach iii) | 0.015 |
| Approach ii) b) v.s. Approach i) | 0.438 |
| Approach ii) b) v.s. Approach ii) | 0.188 |
| Approach ii) b) v.s. Approach iii) | 0.034 |
|  |  |
| Approach i) tissues comparison with Approach ii) tissues | |
| Approach i) a) v.s Approach ii) b) | 0.607 |
| Approach i) b) v.s. Approach ii) a) | 0.641 |
|  |  |
| Full approach comparisons |  |
| Approach i) v.s. Approach ii) | 0.554 |
| Approach i) v.s. Approach iii) | 0.457 |
| Approach ii) v.s. Approach iii) | 0.151 |

**Additional file 3.** Cohen’s *d* value comparing effect of variant quality between approaches.

Approach i) = Non-merged samples (liver and muscle tissue);

Approach i) a) = Non-merged samples (liver);

Approach i) b) = Non-merged samples (muscle);

Approach ii) = Merged samples for low-RFI and merged samples for high-RFI for each tissue (liver and muscle tissue);

Approach ii) a) = Merged samples for low-RFI and merged samples for high-RFI for each tissue (liver tissue);

Approach ii) b) = Merged samples for low-RFI and merged samples for high-RFI for each tissue (muscle tissue);

Approach iii) = Merged samples for low- and high-RFI for both tissues (liver and muscle tissue)
