# Supplementary material for: Development and comparison of RNA-sequencing pipelines for more accurate SNP identification: practical example of functional SNP detection associated with feed efficiency in Nellore beef cattle
Source: BMC Genomics. 2020 Oct 8;21:703. doi: 10.1186/s12864-020-07107-7 (PMC7545862; doi:10.1186/s12864-020-07107-7)
Supplement: Supplementary file 4 — Additional file 4. [file 12864_2020_7107_MOESM4_ESM.docx]

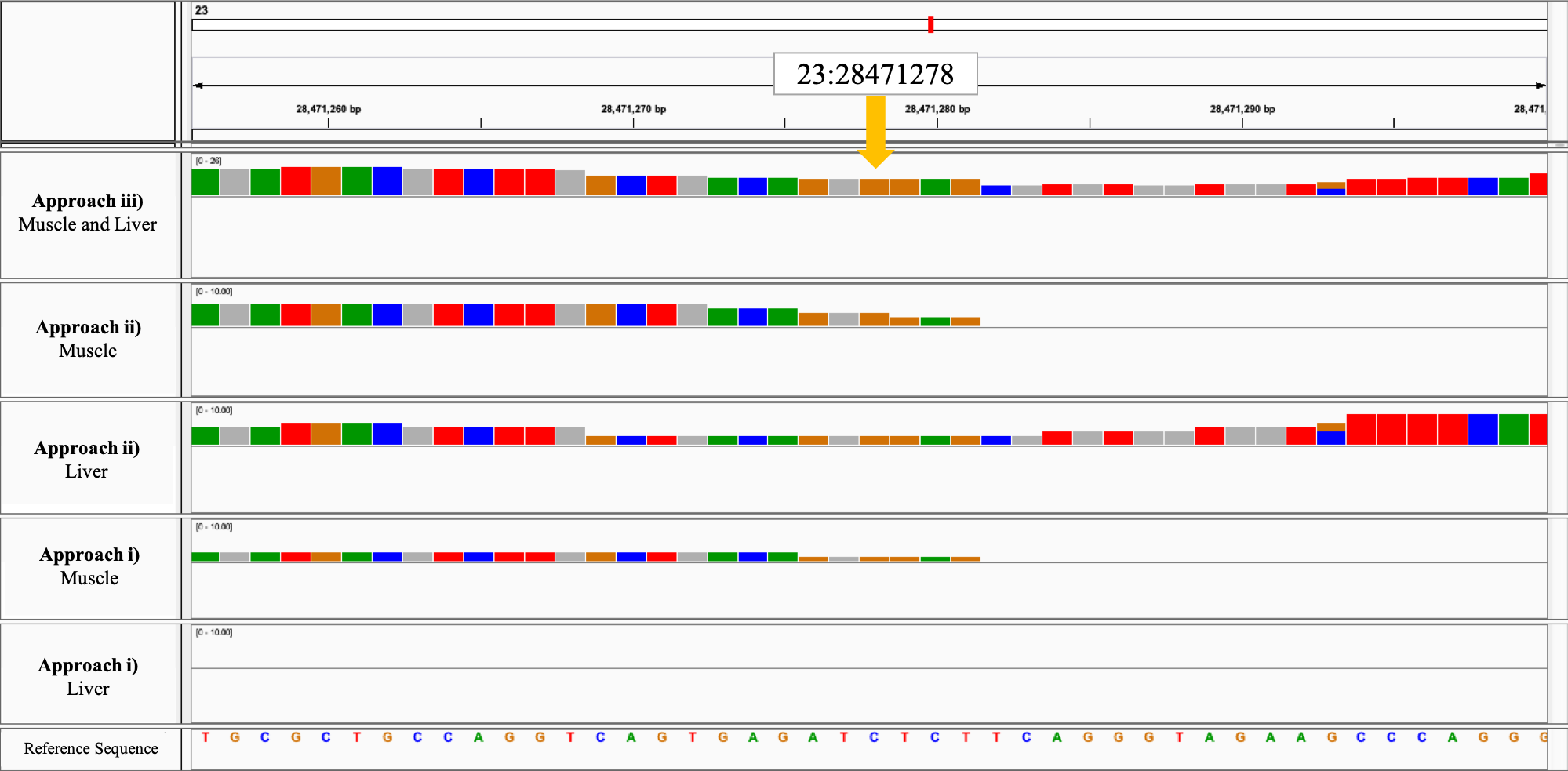
 **Additional file 4.** Visualization of the detection of an example variant (23: 28471278) using Approach iii), which is not detected by Approach i) or Approach ii), and corresponding read depth coverage.

Approach iii) Muscle and Liver: muscle and liver samples merged for low RFI .bam file

Approach ii) Muscle: merged muscle samples for low-RFI .bam file

Approach ii) Liver: merged liver samples for low-RFI .bam file

Approach i) Muscle – non-merged individual muscle sample .bam file (sample accession number: ERS1342445)

Approach i) Liver – non-merged individual liver sample .bam file (sample accession number: ERS579394)

Approach descriptions: i) non-merged samples; ii) merged samples for low-RFI and merged samples for high-RFI for each tissue; iii) merged samples for low- and high-RFI for both tissues

**Legend:** Top numerical row (bp) = base pair position along transcriptome; bottom coloured row (bp letter) = UMD3.1 bovine reference genome (release 94) sequence. Coloured letters: Grey space = nucleotide base matches the reference base, Green = nucleotide base A, Red = nucleotide base T, Blue = nucleotide base C, Orange = nucleotide base G.

**Height of bars along sequence of each approach or sample .bam file** = read depth coverage (higher bar = higher read depth coverage, lower bar = lower read depth coverage)

**Total read count coverage at variant site:** Approach iii) = 10 (alternative allele = G (10), reference allele = C (0)); Approach ii) Muscle = 3 (alternative allele = G (3), reference allele = C (0)); Approach ii) Liver = 2 (alternative allele = G (2), reference allele = C (0)); Approach i) Muscle = 1 (alternative allele = G (1), reference allele = C (0)); Approach i) Liver = 0 (alternative allele = G (0), reference allele = C (0)); Sequence region: Exon.
