# Supplementary material for: Development and comparison of RNA-sequencing pipelines for more accurate SNP identification: practical example of functional SNP detection associated with feed efficiency in Nellore beef cattle
Source: BMC Genomics. 2020 Oct 8;21:703. doi: 10.1186/s12864-020-07107-7 (PMC7545862; doi:10.1186/s12864-020-07107-7)
Supplement: Supplementary file 5 — Additional file 5. [file 12864_2020_7107_MOESM5_ESM.docx]

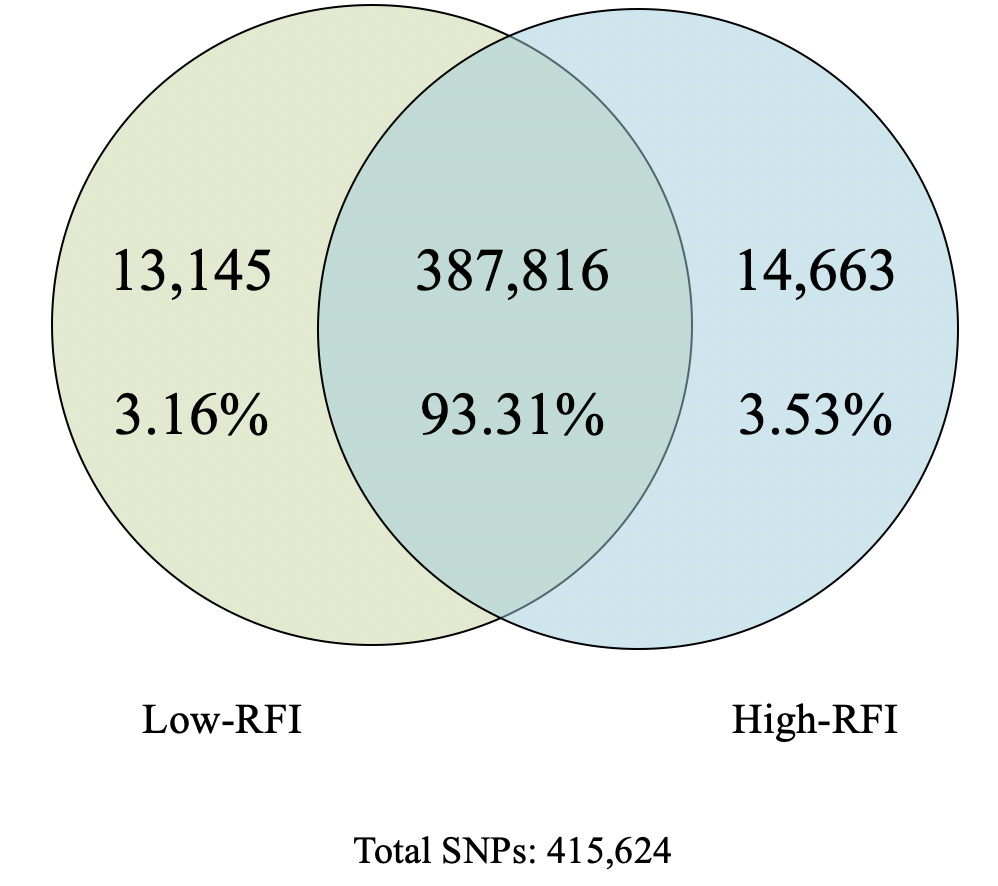


**Additional file 5.** Unique or common SNPs between low- and high-RFI groups using the most optimized RNA-Seq pipeline*.

*Approach iii) merged samples for low- and high-RFI for both tissues.
