# Supplementary material for: Development and comparison of RNA-sequencing pipelines for more accurate SNP identification: practical example of functional SNP detection associated with feed efficiency in Nellore beef cattle
Source: BMC Genomics. 2020 Oct 8;21:703. doi: 10.1186/s12864-020-07107-7 (PMC7545862; doi:10.1186/s12864-020-07107-7)
Supplement: Supplementary file 11 — Additional file 11. [file 12864_2020_7107_MOESM11_ESM.docx]

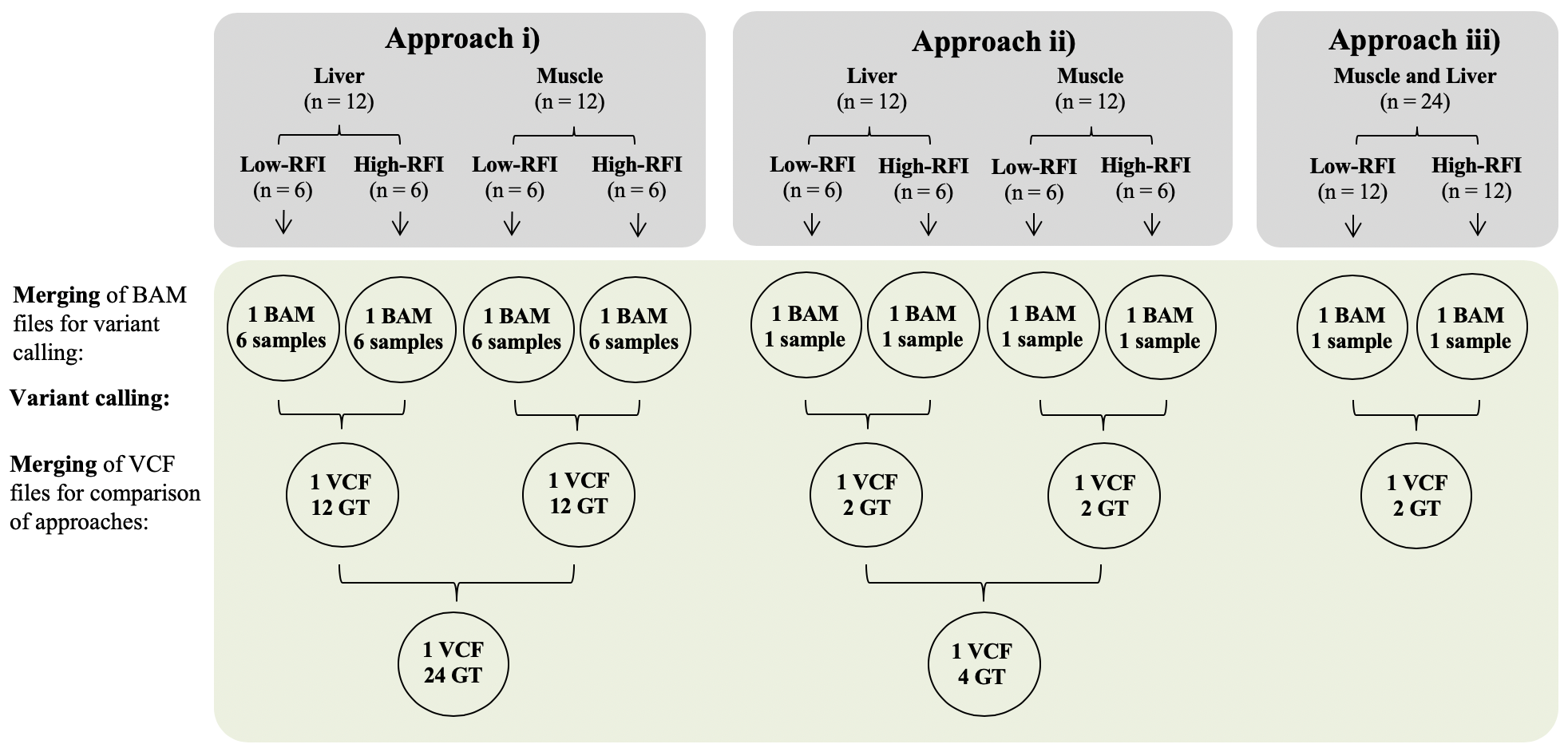


**Additional file 11.** Workflow diagram of sample non-merging and merging strategies for each approach and for approach comparisons.

RFI = residual feed intake; n = sample size; BAM = Binary Alignment Map file; VCF = Variant Calling Format; GT = Genotype.
